# Supplementary material for: Yellow Leaf Disease Resistance and Melanaphis sacchari Preference in Commercial Sugarcane Cultivars
Source: Plants (Basel). 2023 Aug 28;12(17):3079. doi: 10.3390/plants12173079 (PMC10489660; doi:10.3390/plants12173079)
Supplement: Supplementary file 1 [file plants-12-03079-s001.zip › plants-2498723-supplementary.pdf]

Yellow Leaf Disease resistance and *Melanaphis sacchari* preference in commercial sugarcane cultivars

Luiz Eduardo Tilhaqui Bertasello [1], Marcel Fernando da Silva [2], Luciana Rossini Pinto [1,2], Paula Macedo Nóbile [2], Michele Carmo-Sousa [3], Ivan Antônio dos Anjos [2], Dilermando Perecin [1], João Roberto Spotti Lopes [3] and Marcos Cesar Gonçalves [1,4*]

[1]São Paulo State University - UNESP. School of Agricultural and Veterinary Sciences - FCAV, Jaboticabal, São Paulo, 17.884-900 Brazil; luiz.bertasello@unesp.br

[2]Sugarcane Research Centre, Instituto Agronômico de Campinas - IAC, Ribeirão Preto, São Paulo, 14.001-970, Brazil; luciana.rossini@sp.gov.br; marcel.fernsilva@gmail.com

[3]Department of Entomology and Acarology, Escola Superior de Agricultura Luiz de Queiroz (ESALQ), University of São Paulo, Piracicaba 13418-900, Brazil

[4]Crop Protection Research Centre - Instituto Biológico - IB, São Paulo, 04.014-002, Brazil; marcos.goncalves@sp.gov.br

* Correspondence: marcos.goncalves@sp.gov.br (M.C.G.); Tel.: +55-11-50871767

Supplementary Table S1. Variance analysis (ANOVA) for yellow leaf severity of eight sugarcane cultivars across six monthly evaluations in a field assay.

Source	DF	Sum Square	Mean Square	F Value	Pr > F
Cultivar	7	2.6168	0.3738	107.196	<0.001
Block	5	0.0111	0.0022	0.636	0.673
Time	5	0.7455	0.1491	42.755	<0.001
Residuals	270	0.9416	0.0035		
CV (%)			4.132		

Supplementary Table S2. Cycle threshold (Ct) of the reference gene candidates with respective mean Ct, standard deviation, individual and mean efficiencies (eff.), and coefficient of determination (R^2) estimated by RT-qPCR for eight sugarcane cultivars planted in a field trial.

**Reference gene candidate**	**timepoint**	**Ct**	**Individual efficiency**	**R^2**	**Mean Ct ± SD**	**Mean eff. ± SD**
TPI	00 MAP	32.13	1.77	0.99	31.71 ± 0.4	1.85 ± 0.09
TPI	00 MAP	31.35	1.82	0.99		
TPI	00 MAP	31.64	1.70	1.00		
TPI	08 MAP	NA	-	-	-	
TPI	08 MAP	NA	-	-		
TPI	08 MAP	NA	-	-		
TPI	10 MAP	NA	-	-	30.18 ± 0.14	
TPI	10 MAP	30.08	2.01	1.00		
TPI	10 MAP	30.27	1.89	1.00		
TPI	12 MAP	29.47	1.86	1.00	29.29 ± 0.17	
TPI	12 MAP	29.14	1.88	1.00		
TPI	12 MAP	29.26	1.83	1.00		
UBC18	00 MAP	30.68	1.91	1.00	30.7 ± 0.1	1.94 ± 0.04
UBC18	00 MAP	30.61	1.94	1.00		
UBC18	00 MAP	30.81	1.94	1.00		
UBC18	08 MAP	33.23	1.93	0.99	33.4 ± 0.43	
UBC18	08 MAP	33.89	1.92	0.99		
UBC18	08 MAP	33.09	1.91	0.99		
UBC18	10 MAP	31.13	1.96	1.00	30.97 ± 0.14	
UBC18	10 MAP	30.9	1.98	1.00		
UBC18	10 MAP	30.89	2.00	1.00		
UBC18	12 MAP	30.16	1.96	1.00	29.95 ± 0.19	
UBC18	12 MAP	29.84	1.86	1.00		
UBC18	12 MAP	29.84	1.94	1.00		
SAND	00 MAP	28.68	2.04	1.00	28.79 ± 0.1	2.00 ± 0.04
SAND	00 MAP	28.87	1.95	1.00		
SAND	00 MAP	28.82	1.94	1.00		
SAND	08 MAP	30.11	2.02	1.00	30.1 ± 0.04	
SAND	08 MAP	30.13	2.04	1.00		
SAND	08 MAP	30.06	2.03	1.00		
SAND	10 MAP	28.21	1.99	1.00	28.55 ± 0.3	
SAND	10 MAP	28.75	1.96	1.00		
SAND	10 MAP	28.69	2.08	1.00		
SAND	12 MAP	28.22	1.99	1.00	28.5 ± 0.24	
SAND	12 MAP	28.66	1.99	1.00		
SAND	12 MAP	28.62	1.97	1.00		
UK	00 MAP	29.33	1.97	1.00	29.21 ± 0.11	2.02 ± 0.06
UK	00 MAP	29.14	2.00	1.00		
UK	00 MAP	29.15	2.00	1.00		
UK	08 MAP	32.78	1.92	0.99	32.72 ± 0.13	
UK	08 MAP	32.57	2.01	0.99		
UK	08 MAP	32.81	1.99	0.99		
UK	10 MAP	30.88	2.01	1.00	30.98 ± 0.11	
UK	10 MAP	30.94	2.03	1.00		
UK	10 MAP	31.1	2.02	1.00		
UK	12 MAP	28.1	2.13	1.00	28.13 ± 0.03	
UK	12 MAP	28.13	2.10	1.00		
UK	12 MAP	28.17	2.11	1.00		
TUB	00 MAP	26.67	1.98	1.00	26.51 ± 0.16	2.13 ± 0.14
TUB	00 MAP	26.52	2.04	1.00		
TUB	00 MAP	26.34	2.09	1.00		
TUB	08 MAP	29.09	2.11	1.00	29.01 ± 0.09	
TUB	08 MAP	28.91	2.19	1.00		
TUB	08 MAP	29.01	2.55	0.99		
TUB	10 MAP	27.92	2.06	1.00	27.94 ± 0.02	

TUB	10 MAP	27.96	2.17	1.00		
TUB	10 MAP	27.95	2.08	1.00		
TUB	12 MAP	26.26	2.05	1.00	26.11 ± 0.13	
TUB	12 MAP	26.02	2.10	1.00		
TUB	12 MAP	26.05	2.08	1.00		
GAPDH	00 MAP	23.11	2.05	1.00	23.19 ± 0.1	1.99 ± 0.06
GAPDH	00 MAP	23.15	1.96	1.00		
GAPDH	00 MAP	23.3	1.98	1.00		
GAPDH	08 MAP	26.45	1.96	0.99	26.6 ± 0.13	
GAPDH	08 MAP	26.65	2.02	0.99		
GAPDH	08 MAP	26.69	1.85	0.99		
GAPDH	10 MAP	23.18	2.02	1.00	23.21 ± 0.05	
GAPDH	10 MAP	23.18	2.08	1.00		
GAPDH	10 MAP	23.27	1.98	1.00		
GAPDH	12 MAP	22.54	1.96	1.00	22.52 ± 0.09	
GAPDH	12 MAP	22.42	2.00	1.00		
GAPDH	12 MAP	22.59	2.00	1.00		

* NA=Non amplified.

Supplementary Table S3. Cycle threshold (Ct) of the viral target and reference gene (UBC18) with respective mean Ct, standard deviation (SD), individual and mean efficiency, and coefficient of determination (R^2) estimated by RT-qPCR for eight sugarcane cultivars planted in a field trial.

Genotype	timepoint	Ct SCYLV	Individual efficiency	R^2	Mean Ct ± SD	Ct UBC18	Individual efficiency	R^2	Mean Ct ± SD
IACSP95-5094	00 MAP	30.78	1.88	1.00	30.22 ± 0.6	31.08	2.01	1.00	31.17 ± 1.1
IACSP95-5094	00 MAP	30.57	1.94	1.00		32.43	1.93	1.00	
IACSP95-5094	00 MAP	30.51	1.91	1.00		33.02	1.94	1.00	
IACSP95-5094	08 MAP	29.79	1.87	1.00		31.02	2.03	1.00	
IACSP95-5094	08 MAP	29.41	1.95	1.00		31.02	2.03	1.00	
IACSP95-5094	08 MAP	29.36	1.86	1.00		31.62	2.00	0.99	
IACSP95-5094	10 MAP	30.20	1.99	1.00		31.20	2.02	1.00	
IACSP95-5094	10 MAP	30.96	2.11	0.99		32.01	2.08	0.99	
IACSP95-5094	10 MAP	31.26	1.91	1.00		31.68	1.96	1.00	
IACSP95-5094	12 MAP	29.97	1.90	1.00		29.69	1.98	1.00	
IACSP95-5094	12 MAP	30.00	1.92	1.00		29.57	1.97	1.00	
IACSP95-5094	12 MAP	29.80	1.94	1.00		29.67	1.96	1.00	
IACCTC05-2562	00 MAP	37.44	1.89	1.00	34.38 ± 4.11	30.25	2.00	1.00	31.71 ± 3.41
IACCTC05-2562	00 MAP	38.51	1.91	1.00		29.95	2.05	1.00	
IACCTC05-2562	00 MAP	37.97	1.05	0.39		32.02	1.97	1.00	
IACCTC05-2562	08 MAP	29.86	1.87	1.00		28.47	1.96	1.00	
IACCTC05-2562	08 MAP	29.22	1.99	1.00		28.64	1.94	1.00	
IACCTC05-2562	08 MAP	30.41	1.96	1.00		28.73	1.99	1.00	
IACCTC05-2562	10 MAP	NA	1.87	1.00		30.66	2.04	1.00	
IACCTC05-2562	10 MAP	NA	1.84	1.00		30.91	2.02	1.00	
IACCTC05-2562	10 MAP	38.26	1.94	1.00		29.64	1.98	1.00	
IACCTC05-2562	12 MAP	33.36	1.95	1.00		36.49	1.85	1.00	
IACCTC05-2562	12 MAP	NA	1.00	0.00		36.98	1.96	1.00	
IACCTC05-2562	12 MAP	NA	1.00	0.00		37.78	2.19	1.00	
IACSP01-5503	00 MAP	36.35	1.89	1.00	36.49 ± 0.85	31.52	1.95	1.00	32.67 ± 2.58
IACSP01-5503	00 MAP	35.90	1.84	1.00		31.49	1.99	1.00	
IACSP01-5503	00 MAP	37.20	1.92	1.00		31.68	1.95	1.00	
IACSP01-5503	08 MAP	NA	1.12	0.87		37.17	2.01	1.00	
IACSP01-5503	08 MAP	NA	1.03	0.94		37.10	1.88	1.00	
IACSP01-5503	08 MAP	NA	1.00	0.00		36.44	1.99	1.00	
IACSP01-5503	10 MAP	35.32	1.88	1.00		31.42	2.00	0.99	
IACSP01-5503	10 MAP	36.57	1.90	1.00		31.49	2.05	1.00	

IACSP01-5503	10 MAP	36.22	1.90	1.00		31.59	2.13	0.98	
IACSP01-5503	12 MAP	37.90	1.83	1.00		30.74	2.04	1.00	
IACSP01-5503	12 MAP	NA	1.77	1.00		30.73	1.91	1.00	
IACSP01-5503	12 MAP	NA	1.00	0.00		30.66	1.99	1.00	
IACSP96-7569	00 MAP	34.93	1.89	1.00	32.33 ± 2.49	31.46	1.92	1.00	32.43 ± 1.01
IACSP96-7569	00 MAP	36.55	1.90	1.00		31.50	1.92	1.00	
IACSP96-7569	00 MAP	35.86	1.92	1.00		31.00	1.94	1.00	
IACSP96-7569	08 MAP	32.88	1.83	1.00		33.64	2.00	1.00	
IACSP96-7569	08 MAP	32.88	1.88	1.00		33.91	1.98	1.00	
IACSP96-7569	08 MAP	33.10	1.85	1.00		34.05	1.96	1.00	
IACSP96-7569	10 MAP	30.79	1.90	1.00		32.47	1.95	1.00	
IACSP96-7569	10 MAP	31.18	1.87	1.00		32.56	1.97	1.00	
IACSP96-7569	10 MAP	31.49	1.89	1.00		32.79	1.97	1.00	
IACSP96-7569	12 MAP	29.17	1.84	1.00		32.12	1.97	1.00	
IACSP96-7569	12 MAP	29.55	1.89	1.00		31.80	1.97	1.00	
IACSP96-7569	12 MAP	29.53	1.90	1.00		31.80	1.99	1.00	
IACBIO-266	00 MAP	26.41	1.87	1.00	34.24 ± 6.16	30.22	2.00	1.00	30.65 ± 0.75
IACBIO-266	00 MAP	26.79	1.90	1.00		30.02	1.98	1.00	
IACBIO-266	00 MAP	27.02	1.88	1.00		30.01	1.96	1.00	
IACBIO-266	08 MAP	40.00	1.89	1.00		30.94	1.95	1.00	
IACBIO-266	08 MAP	40.00	1.86	1.00		30.95	2.30	1.00	
IACBIO-266	08 MAP	40.00	1.90	1.00		31.01	1.95	1.00	
IACBIO-266	10 MAP	29.97	1.86	1.00		31.74	1.93	1.00	
IACBIO-266	10 MAP	30.30	1.87	1.00		31.83	1.94	1.00	
IACBIO-266	10 MAP	30.33	1.87	1.00		31.35	1.95	1.00	
IACBIO-266	12 MAP	40.00	1.92	1.00		29.78	1.95	1.00	
IACBIO-266	12 MAP	40.00	1.84	1.00		29.73	1.96	1.00	
IACBIO-266	12 MAP	40.00	1.92	1.00		30.16	1.93	1.00	
IACSP01-3127	00 MAP	26.16	1.89	1.00	28.47 ± 1.75	31.29	1.93	1.00	31.1 ± 0.51
IACSP01-3127	00 MAP	26.52	1.92	1.00		31.06	1.94	1.00	
IACSP01-3127	00 MAP	26.54	1.90	1.00		30.99	1.93	1.00	
IACSP01-3127	08 MAP	NA	1.04	0.85		NA	1.00	0.00	
IACSP01-3127	08 MAP	NA	1.01	0.81		NA	1.05	0.71	
IACSP01-3127	08 MAP	NA	1.01	0.80		NA	1.05	0.06	
IACSP01-3127	10 MAP	28.43	1.75	1.00		31.67	1.99	1.00	
IACSP01-3127	10 MAP	28.56	1.92	1.00		31.55	1.97	1.00	
IACSP01-3127	10 MAP	28.75	1.93	1.00		31.71	1.96	1.00	
IACSP01-3127	12 MAP	30.22	1.89	1.00		30.24	2.03	1.00	
IACSP01-3127	12 MAP	30.50	1.90	1.00		30.88	1.95	1.00	
IACSP01-3127	12 MAP	30.53	1.91	1.00		30.54	1.90	1.00	
IACSP95-5000	00 MAP	36.75	1.84	0.99	30.84 ± 3.69	35.77	1.80	1.00	33.08 ± 1.49
IACSP95-5000	00 MAP	37.59	1.73	0.99		35.32	1.83	0.99	

IACSP95-5000	00 MAP	35.82	1.81	1.00		34.93	1.86	0.99	
IACSP95-5000	08 MAP	28.41	1.99	1.00		32.37	2.01	1.00	
IACSP95-5000	08 MAP	28.56	2.06	1.00		33.23	1.91	1.00	
IACSP95-5000	08 MAP	27.85	1.91	1.00		33.36	1.96	1.00	
IACSP95-5000	10 MAP	30.66	1.91	1.00		32.19	1.93	1.00	
IACSP95-5000	10 MAP	27.52	2.05	1.00		32.75	1.93	1.00	
IACSP95-5000	10 MAP	30.70	1.95	1.00		32.38	1.93	1.00	
IACSP95-5000	12 MAP	28.88	1.92	1.00		31.62	1.88	1.00	
IACSP95-5000	12 MAP	28.53	1.99	1.00		31.53	1.97	1.00	
IACSP95-5000	12 MAP	28.82	1.92	1.00		31.54	1.91	1.00	
SP71-6163	00 MAP	26.66	1.93	1.00	27.23 ± 1.35	31.70	1.90	1.00	31.1 ± 0.92
SP71-6163	00 MAP	25.81	1.87	1.00		31.77	1.91	1.00	
SP71-6163	00 MAP	24.94	1.93	1.00		31.17	1.89	1.00	
SP71-6163	08 MAP	27.29	2.02	1.00		30.00	1.91	1.00	
SP71-6163	08 MAP	25.81	1.91	1.00		29.87	1.93	1.00	
SP71-6163	08 MAP	26.28	2.02	0.99		30.24	1.88	1.00	
SP71-6163	10 MAP	27.41	2.00	1.00		32.50	1.94	1.00	
SP71-6163	10 MAP	28.31	1.98	1.00		31.62	1.94	1.00	
SP71-6163	10 MAP	27.75	2.00	1.00		32.44	1.92	1.00	
SP71-6163	12 MAP	28.24	1.99	1.00		31.04	1.89	1.00	
SP71-6163	12 MAP	29.01	1.97	1.00		30.54	1.91	1.00	
SP71-6163	12 MAP	29.20	2.01	1.00		30.27	1.89	1.00	
**Mean eff. ± SD**			**1.90 ± 0.11**				**1.96 ± 0.07**		

* NA=Non amplified.

Supplementary Table S4. Melting temperature and detection of 181 bp RT-qPCR products of the SCYLV capsid protein for eight sugarcane cultivars in each sampling time point, namely 0, 8, 10, and 12 months after planting (MAP).

		Technical replicate			
Genotype	Sampling timepoint	1	2	3	Pos/Neg
IACSP95-5094	0 MAP	82.0	82.0	82.5	+
IACSP95-5094	8 MAP	82.5	82.5	82.5	+
IACSP95-5094	10 MAP	82.5	82.5	82.5	+
IACSP95-5094	12 MAP	82.5	82.5	82.5	+
IACCTC05-2562	0 MAP	83.0	83.0	74.5	+
IACCTC05-2562	8 MAP	83.0	82.5	82.5	+
IACCTC05-2562	10 MAP	83.0	83.5	83.0	+
IACCTC05-2562	12 MAP	83.0	84.0	85.5	+
IACSP01-5503	0 MAP	82.5	82.5	82.5	+
IACSP01-5503	8 MAP	63.5	63.5	63.5	–
IACSP01-5503	10 MAP	82.5	82.5	82.5	+
IACSP01-5503	12 MAP	83.0	83.0	66.0	+
IACSP96-7569	0 MAP	82.0	82.0	82.5	+
IACSP96-7569	8 MAP	82.5	82.5	82.5	+
IACSP96-7569	10 MAP	82.5	82.5	82.5	+
IACSP96-7569	12 MAP	82.5	82.5	82.5	+
IACBIO-266	0 MAP	82.5	82.5	82.5	+
IACBIO-266	8 MAP	83.0	83.0	83.0	+
IACBIO-266	10 MAP	82.5	82.5	82.5	+
IACBIO-266	12 MAP	83.5	83.0	83.5	+
IACSP01-3127	0 MAP	82.5	82.5	82.5	+
IACSP01-3127	8 MAP	63.5	63.5	63.5	–
IACSP01-3127	10 MAP	81.5	82.5	82.5	+
IACSP01-3127	12 MAP	82.5	82.0	82.5	+
IACSP95-5000	0 MAP	83.0	82.5	82.5	+
IACSP95-5000	8 MAP	82.5	82.5	82.5	+
IACSP95-5000	10 MAP	82,5	82.5	82.5	+
IACSP95-5000	12 MAP	82.5	82.5	82.5	+
SP71-6163	0 MAP	83.0	82.5	82.5	+
SP71-6163	8 MAP	83.0	82.5	82.5	+
SP71-6163	10 MAP	82.5	82.5	82.5	+
SP71-6163	12 MAP	82.5	82.5	82.5	+

RT-qPCR melting temperature of 82.5± 1.0 °C are positive to the 181 bp fragment of the SCYLV capsid protein in the genotypes. (+) SCYLV positive samples and (–) negative samples.

Supplementary Table S5. Analyses of candidate reference genes by the NormFinder algorithm.

Gene name	Stability value	Best Gene	Best combination of two genes
Triosephosphate isomerase (TPI)	0.379		
Ubiquitin-conjugating enzyme 18 gene (UBC18)	0.224	0.224	
SAND protein family (SAND)	0.573		
Uridylate kinase (UK)	0.458		0.168
TUBULIN (TUB)	0.284		0.168
Glyceraldehyde-3-phosphate dehydrogenase (GAPDH)	0.354		

Supplementary Table S6. One-way variance analysis (ANOVA) for the number apterous adult non-viruliferous *Melanaphis sacchari* for the timepoints of 0.5, 1, 3, 6, and 24 h after release, in a free-choice bioassay with eight sugarcane cultivars.

		0.5 h			
Source	DF	Sum of Squares	Mean Square	F Value	Pr > F
Cultivar	7	0.55452426	0.07921775	0.92	0.4926
Error	120	10.31706417	0.08597553		
CV (%)			15.78578		
		1 h			
Source	DF	Sum of Squares	Mean Square	F Value	Pr > F
Cultivar	7	0.59436927	0.08490990	0.76	0.6243
Error	120	13.45939532	0.11216163		
CV (%)			17.47641		
		3 h			
Source	DF	Sum of Squares	Mean Square	F value	Pr > F
Cultivar	7	0.53137914	0.07591131	0.53	0.8089
Error	120	17.12701808	0.14272515		
CV (%)			19.35157		
		6 h			
Source	DF	Sum of Squares	Mean Square	F value	Pr > F
Cultivar	7	0.35090945	0.05012992	0.38	0.9121
Error	120	15.80052990	0.13167108		
CV (%)			18.88871		
		24 h			
Source	DF	Sum of Squares	Mean Square	F value	Pr > F
Cultivar	7	0.79978061	0.11425437	0.73	0.6502
Error	120	18.88645032	0.15738709		
CV (%)			20.26387		

Supplementary table S7. Primer pairs sequences and amplicon size (A) and melting temperatures (Tm), estimated by RT-qPCR, of six genes selected for stability assessment across four sampling timepoints of sugarcane under sugarcane yellow leaf virus (SCYLV) infection, namely, triosephosphate isomerase (TPI), Uridylate kinase (UK), SAND protein family (SAND), Ubiquitin-conjugating enzyme 18 gene (UBC18), glyceraldehyde-3-phosphate dehydrogenase (GAPDH), and TUBULIN (TUB).

Primers	Sequence F (5′-3′)	Sequence R (5′-3′)	A (bp)	Tm (ºC)
TPI[a]	CAATGACTGGAGCAACGTAG	GTAACAGAGCCTCCGTAGAT	200	84.5
UK[b]	GCAATCTAAGGGACATAATAAAGGTG	AATCGAATTGCCTACTGATATGTTG	140	78.5
SAND[b]	CTGTGTGTAAGTTGATATGTCTATGTTG	CAACAACGATGGTACTGCCT	94	77.5
UBC18[b]	GCCTGTCAGCCTTCCTTAC	GGTAAGCTTCGCAAATCCAATAG	100	79.5
GAPDH[c]	TTGGTTTCCACTGACTTCGTT	CTGTAGCCCCACTCGTTGT	122	84.5
TUB[c]	CTCCACATTCATCGGCAACTC	TCCTCCTCTTCTTCCTCCTCG	237	87.0

a: Primer pairs sequences reported by Zhu et al. (2010); b: Primer pairs sequences reported Silva et al. (2019); c: Primer pairs sequences reported by Andrade et al. (2017).

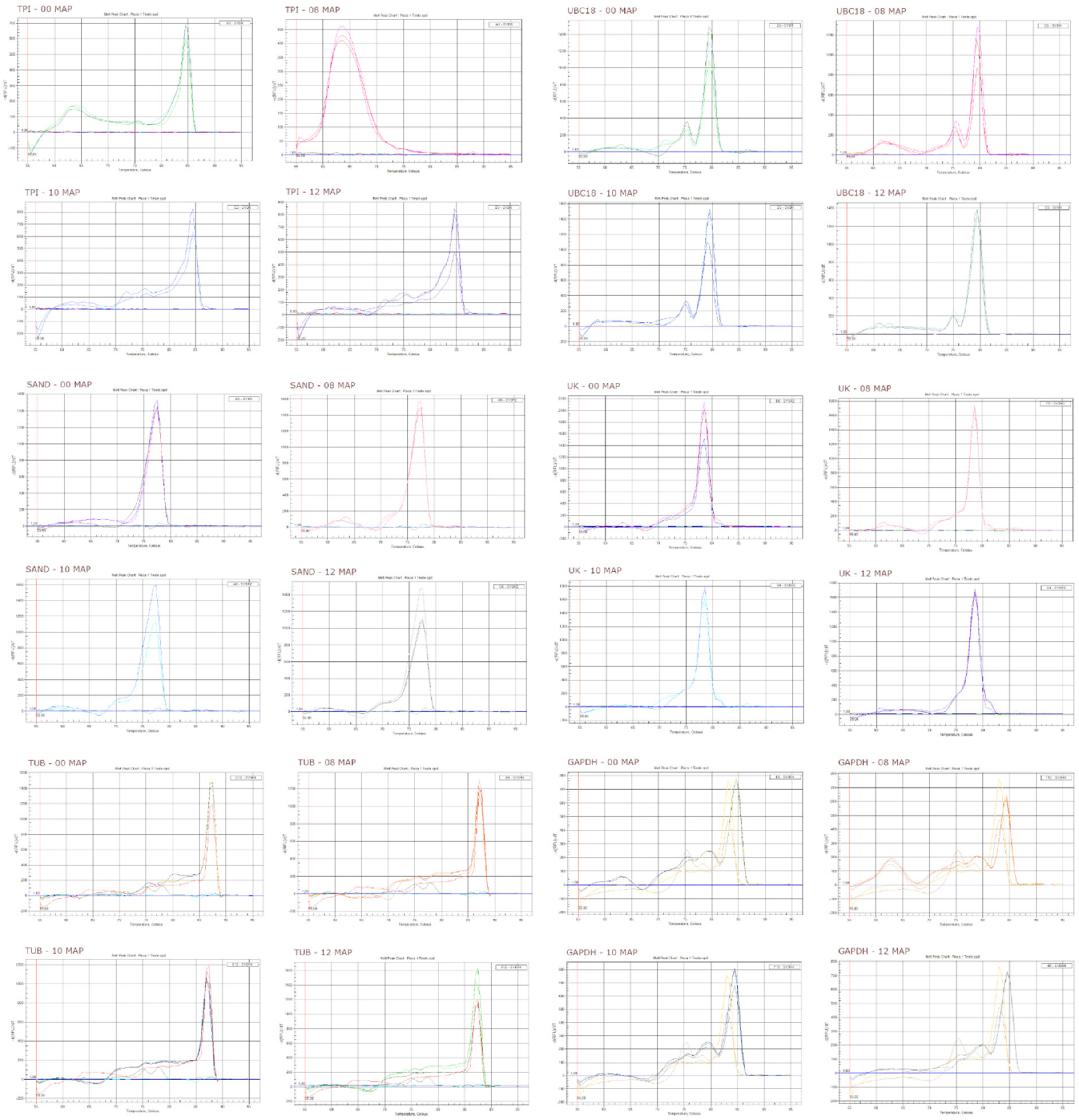

Supplementary figure S1. Dissociation curve of six genes selected for stability assessment across four cDNA pools, each representing the sampling times of 0, 8, 10, and 12 months after planting (MAP) and comprising eight sugarcane cultivars under sugarcane yellow leaf virus (SCYLV) infection. The candidate reference genes were triosephosphate isomerase (TPI), Uridylate kinase (UK), SAND protein family (SAND), Ubiquitin-conjugating enzyme 18 gene (UBC18), glyceraldehyde-3-phosphate.

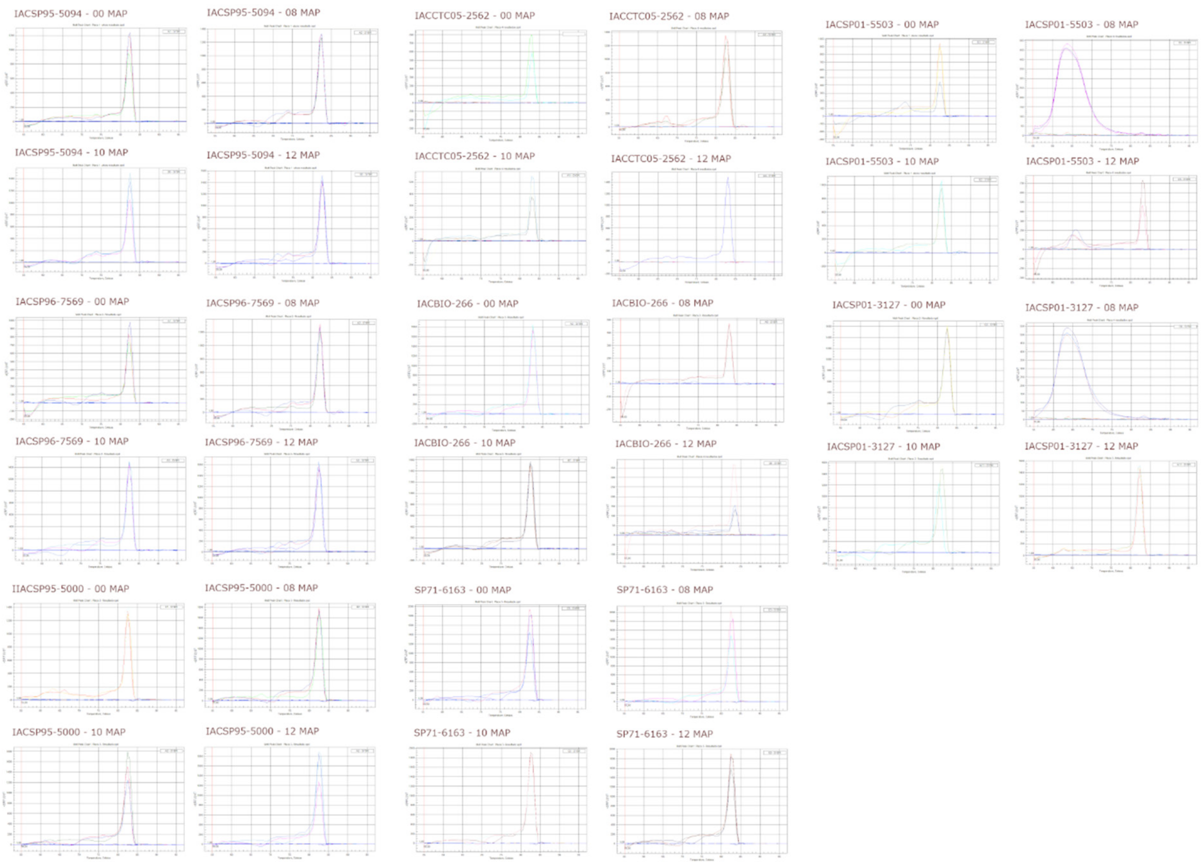

Supplementary figure S2. Dissociation curve of the target gene, i.e., 181 bp fragment of ScYLV capsid protein, across cDNA pools from eight sugarcane cultivars under sugarcane yellow leaf virus (SCYLV) infection and four sampling timepoints, namely 0, 8, 10, and 12 months after planting (MAP).

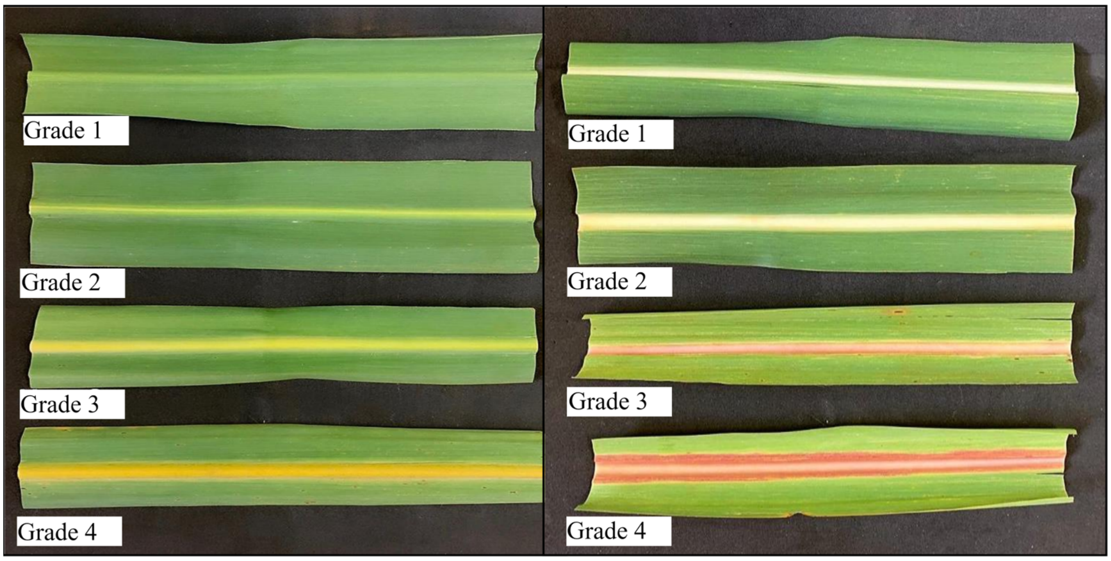

Supplementary figure S3. Diagrammatic scale for SCYLV symptoms intensity. Grade 1: green leaf without symptoms; Grade 2: slight yellowing of the midrib and leaf blade; Grade 3: intense yellowing of the midrib and partial yellowing of the leaf blade; Grade 4: intense yellowing of the midrib and leaf blade.

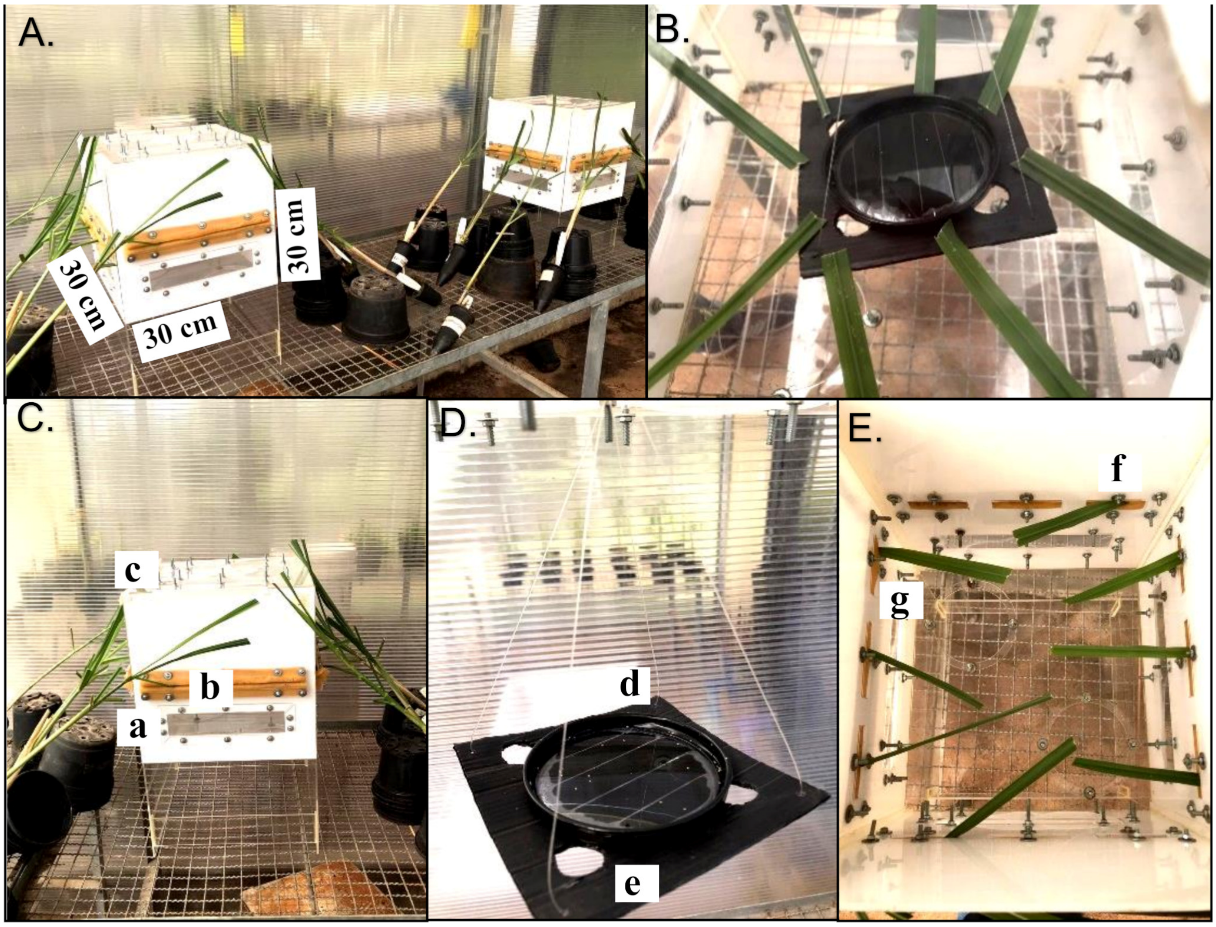

Supplementary figure S4. Free-choice bioassay aiming to evaluate the settling behavior of *Melanaphis sacchari* among eight sugarcane cultivars. (A) Acrylic cages (30 cm × 30 cm × 30 cm), each comprising individual leaves of plantlets from eight sugarcane cultivars. Plantlets were maintained in stiff plastic tubes and randomly distributed. (B) Individual leaves of eight sugarcane cultivars placed in a squared black platform within an acrylic cage. The squared black platform supported the circular acrylic arena where fifty apterous adult non-viruliferous were released. (C) Side view of an acrylic cage with side openings (4 x 20 cm) sealed with "voile" fabric for ventilation (a) and openings through which individual leaves were inserted (b). On top of the acrylic cage (c) there are openings (4 x 20 cm) also sealed with "voile" fabric; for a top view of a closed acrylic cage, see Ramos et al. [64]. (D) Circular arena (d) supported by a squared black platform (e), which was fixed on the roof of the acrylic cage. (E) Internal side view showing individual leaves inserted through side openings, e.g., (f) and (g).
